# Resveratrol Targets AKT1 to Inhibit Inflammasome Activation in Cardiomyocytes Under Acute Sympathetic Stress

**DOI:** 10.3389/fphar.2022.818127

**Published:** 2022-02-17

**Authors:** Rui Wang, Yanming Wang, Jimin Wu, Yanli Guo, Han Xiao, Youyi Zhang, Ketao Ma

**Affiliations:** ^1^ Key Laboratory of Xinjiang Endemic and Ethnic Diseases, Ministry of Education, Shihezi University School of Medicine, Shihezi, China; ^2^ NHC Key Laboratory of Prevention and Treatment of Central Asia High Incidence Diseases, First Affiliated Hospital, Shihezi University School of Medicine, Shihezi, China; ^3^ Department of Physiology, Shihezi University School of Medicine, Shihezi, China; ^4^ Department of Cardiology and Institute of Vascular Medicine, Peking University Third Hospital, NHC Key Laboratory of Cardiovascular Molecular Biology and Regulatory Peptides, Key Laboratory of Molecular Cardiovascular Science, Ministry of Education, Beijing Key Laboratory of Cardiovascular Receptors Research, Beijing, China

**Keywords:** resveratrol, AKT1, NLRP3 inflammasome, network pharmacology, molecular docking

## Abstract

Resveratrol shows promizing anti-inflammatory effects in recent clinical trials, however its function in cardiovascular patients remains conflicting, suggesting there may be new mechanisms underlying its cardioprotective activity. Acute sympathetic stress induces early activation of the NLR family, pyrin domain containing 3 (NLRP3) inflammasome in cardiomyocytes as a critical step for triggering cardiac inflammation. Thus, this study explored targets of resveratrol activity involved in the inhibition of early inflammasome activation in cardiomyocytes following acute sympathetic stress. Network pharmacology was used to analyze common candidate targets in the sympathetic stress pathway, resveratrol activity, and myocardial inflammation and showed the Phosphoinositol 3—kinase (PI3K)/serine threonine protein kinase (Akt) signaling pathway and the target AKT1 may play a critical role. Molecular docking provided support for potential binding of resveratrol on AKT1. Furthermore, the effect of resveratrol on AKT1 activation was determined in cardiomyocytes. resveratrol dose-dependently inhibited AKT1 activation after activation of β-adrenoceptor. The AKT1 inhibitor A-674563 suppressed the activation of the NLRP3 inflammasome in cardiomyocytes following β-adrenoceptor activation, suggesting that AKT1 is a critical regulator molecule upstream of the NLRP3 inflammasome. Consistently, treatment with resveratrol suppressed β-adrenoceptor-mediated NLRP3 inflammasome activation in both cardiomyocytes and mouse hearts, as well as the resultant cardiac inflammation. In conclusion, resveratrol targets AKT1 to inhibit NLRP3 inflammasome activation in cardiomyocytes and cardiac inflammation following acute sympathetic stress. AKT1 is an important target of resveratrol, which should be considered as a treatment option for cardiovascular patients, especially those at risk of acute sympathetic stress.

## Introduction

Inflammation plays a critical role in the progression of various cardiovascular diseases and anti-inflammatory strategies have been shown to have promising therapeutic potential. Resveratrol, a small-molecule monomer polyphenol compound (3,4 9,5-trihydroxy-trans-stilbene) found abundantly in grapes and red wine (Novelle et al., 2015; Lin et al., 2019) is a multi-target drug that shows both cardioprotective and anti-inflammatory effects on many cardiovascular diseases in preclinical studies (Sung and Dyck, 2015; Bernal-Ramírez et al., 2021; Zhang et al., 2021). Some clinical trials have also shown its anti-inflammatory activity (Berman et al., 2017; Singh et al., 2019), which was further confirmed by a meta-analysis evaluating its relationship with C-reactive protein (Gorabi et al., 2021). However, the results of clinical trials on the cardiac protective effect of resveratrol remain conflicting (Dyck et al., 2019), which suggests its role in the heart may involve new unidentified targets and may be dependent on specific pathologic conditions.

Sympathetic nervous system (SNS) overactivation is a main pathologic condition that contributes to many cardiovascular diseases, such as myocardial infarction and heart failure (Dünser and Hasibeder, 2009; Boland et al., 2015; Kong et al., 2017). Resveratrol was shown to suppress cardiac inflammation in mice after sympathetic activation in a recent study (Nie et al., 2020), suggesting its protective effects on the heart under SNS overactivation. Acute sympathetic stress has been reported to induce cardiac inflammation by activating the pyrin domain containing 3 (NLRP3) inflammasome. Although it also plays a critical role in immune cells, the inflammasome is early activated in cardiomyocytes following acute sympathetic stress, which triggers cardiac inflammation. (Xiao et al., 2018; Cao et al., 2021). However, it remains unknown whether and how resveratrol inhibits the early inflammasome activation in cardiomyocytes following acute sympathetic stress.

We screen potential targets of resveratrol in inflammasome inhibition using network pharmacological analysis. Network pharmacology was introduced in the post-genomic era to deal with the increasing number of potential targets discovered by the rapid development of pharmacological technology (Recanatini and Cabrelle, 2020). It uses a computer-aided target prediction algorithm to analyze structural information of drug targets and potential targets. Herein, we used network pharmacological analysis to screen the targets of resveratrol, sympathetic stress, and myocardial inflammation with network pharmacological analysis, to identify the novel targets of the anti-inflammatory activity of resveratrol following acute sympathetic stress. Further, this study investigates the role of resveratrol in inflammasome activation in cardiomyocytes after sympathetic stress. The study will be helpful in defining the translational application of resveratrol in clinical therapy.

## Materials and Methods

### Network Pharmacology Approach

This study used the TCMSP database (http://tcmspw.com/tcmsp.php), the SWISS target prediction database (http://swisstargetprediction.ch/) (Daina et al., 2019), the SEA database (http://sea.bkslab.org), and the QSAR database (quantitative structure activity relationships) to predict potential resveratrol targets. The target genes were then transformed into UniProt gene ID with the UniProt database (https://www.Unitprot.org/). The targets of resveratrol were then combined and duplicate items were deleted. The targets for sympathetic stress and myocardial inflammation were obtained in the gene cards database by searching with “sympathetic stress” and “myocardial inflammation,” respectively. Targets were also transformed into UniPort gene IDs using the UniProt database.

These targets were analyzed with Venn diagram analysis (http://bioinformatics.psb.ugent.be/webtools/Venn/). The common target genes were further analyzed with the String database (https://string-db.org). Hone sapiens was selected as the species, and the reliability was >0.4. Finally, the protein-protein interaction (PPI) network of resveratrol-sympathetic-myocarditis was constructed. The common targets were sorted according to the parameter values of the protein-protein relationship (degree, betweenness, and closeness). The results of the PPI data were exported into Cytoscape_V3.6 software (Szklarczyk et al., 2019) to determine the common targets hub genes of resveratrol, sympathetic stress, and myocardial inflammation. These target genes were then transformed into ensemble IDs with the Ensembl database tools (http://asia.ensembl.org/index.html) and submitted for KEGG pathway analysis (Zhou et al., 2019).

### Molecular Docking

To clarify whether resveratrol directly targets AKT1, the molecular docking method was used to evaluate the interaction between resveratrol and the key core target. The three-dimensional (3D) crystal structure of AKT1 (PDB ID: 4EJN) was obtained from the Protein Data Bank (PDB). The two-dimensional (2D) SDF format of resveratrol and of A-674563 were downloaded from PubChem. Docking experiments were performed using CDOCKER with receptor-ligand interactions. The -CDOCKER_INTERACTION_ENERGY value was determined to evaluate bonding. The binding results were visualized as 3D and 2D diagrams using Discovery Studio™ 2.5 (DS; Accelrys sofware Inc., San Diego, CA, United States).

### Animals

All animal experimental protocols were approved by the Committee of Peking University on the Ethics of Animal Experiments (LA2018144). The study was conducted in accordance with the use of laboratory animals published by the United States National Institutes of Health (NIH Publication No. 85-23, revised 2011) and the guidelines of the Peking University Health Science Center. Male 10–12 week old C57BL/6J mice (weight 25–30 g) and neonatal C57BL/6J mice (0–3 days) were purchased from *Beijing Weitong Lihua Experimental Animal Technology Co*., *Ltd*., and the experimental animal center of the Peking University Health Science Center. All mice were raised in the experimental animal center (environment: relative humidity 65%, room temperature 23°C, alternating 12 h light and dark cycles).

A total of 24 mice were randomly divided into four groups with six mice in each group: Control group (saline injection group), isoproterenol group (isoproterenol treatment group), isoproterenol + resveratrol group (resveratrol pretreatment + isoproterenol treatment group), and resveratrol group (resveratrol alone treatment group). The isoproterenol + resveratrol group and the resveratrol group received resveratrol (20 mg/(kg•d)) by intragastric gavage for 4 days. On the fourth day, the isoproterenol group and the isoproterenol + resveratrol group received a single subcutaneous (sc) injection of isoproterenol (5mg/kg), the selective agonist of β-adrenoceptors. On the 7th day, the mice were euthanized with overdose sodium pentobarbital. Cardiac samples were collected as described previously (Cao et al., 2021).

### Cell Culture

Neonatal mouse cardiomyocytes (NMCM) from neonatal C57BL/6J mice (0–3 days) were isolated by enzymatic disassociation according to the neonatal heart dissociation kit manual (Miltenyi Biotec, Germany). The isolated cardiomyocytes were cultured in Dulbecco’s modified Eagle medium (DMEM) containing 15% fetal bovine serum (FBS) and high glucose for 2 h, then the culture medium rich in cardiomyocytes was inoculated in a six-well plate (5 × 10^5^ cells per well), and 100 μmol/L bromodeoxyuridine (Sigma) was added to prevent fibroblast proliferation. After further culture for 36–48 h, the cells were starved in the serum-free medium for 3–4 h before drugs and intervention treatment.

### Immunohistochemistry

MAC-3^+^ immunohistochemical staining of mouse heart tissue was used to assess macrophage infiltration. Mouse heart tissue was washed with cold phosphate buffered saline, fixed with 4% paraformaldehyde for 6–8 h, and embedded in paraffin. Cardiac tissue sections (5 μmol thick) were stained with an antibody against the macrophage specific marker MAC-3 (BD Biosciences, rat derived, dilution 1:200). The sections were imaged with a NanoZoomer-SQ digital slide scanner (Hamamatsu Photonics, Shizuoka, Japan). To evaluate macrophage infiltration, 10 fields were randomly selected from each section, The percentage of MAC-3 positive staining (brown stained part) in the whole myocardial area was calculated by Image Pro Plus 6.0 image analysis software. Immunohistochemical positive staining (brown staining) was evaluated using GraphPad Prism software (version 8.0.1, GraphPad Software Inc., San Diego, CA, United States).

### Western Blotting Analysis

Protein samples were separated by 12% SDS polyacrylamide gel electrophoresis (30–40 μg), and transferred to a nitrocellulose membrane (NC membrane), which was blocked with 5% skim milk or 5% bovine serum albumin at room temperature for 1 h, and incubated overnight on a shaker at 4°C with antibodies: NLRP3 (1:500, CST); Pro-caspase-1 (1:1,000, CST); Caspase-1(p20) (1:1,000, Sigma); Pro-interleukin (IL)-18 (1:1,000, Abcam); Cleaved-IL-18 (1:1,000, MBL); GAPDH (1:1000 CST); Akt1 (1:3,000, CST); P-Akt1 (1:5,000, CST); SIRT1 (1:5,000, CST). Then the membrane was incubated with the corresponding secondary antibody at room temperature for 2 h, visualized with Immobilon Western Chemiluminescent HRP Substrate (Millipore Corporation, St. Burlington, MA, United States). The Syngene GeneGnome-XRQ-NPC imager (Gene Company, Shanghai, China) was used to obtain the blot images, and the band gray value was analyzed using Image 6.0 software.

### ELISA

The cytokines of macrophage chemokine protein-1 (MCP-1), macrophage chemokine protein-5 (MCP-5), IL-6, and tumor necrosis factor-α (TNF-α) in heart tissue were detected by the ELISA kit (R&D) as previously described (Xin et al., 2020). Briefly, 50 μmol whole tissue homogenate, or protein 50 μl standard and control was added and incubated at room temperature for 2 h. After washing with washing buffer, 100 μl substrate reaction solution was added to each well (the surface is covered with tin foil to avoid light). After incubation at room temperature for 30 min, 100 μl reaction termination solution was added to each well and the OD value was measured at 450 and 570 nm using a microplate reader. The cytokine concentrations were adjusted the protein concentrations of each sample.

### *In vitro* AKT1 Kinase Activity

For *in vitro* AKT1 kinase activity assays, recombinant active AKT1 kinase (Abcam) and GSK-3β (SinoBiological) substrates were added to 30 μl kinase buffer. The AKT1 kinase activity was indicated by the phosphorylation of GSK-3β. Kinase reactions were performed at 30°C for 30 min, and the samples were heated to 100°C for 5 min followed by the addition of SDS loading buffer. Reaction products were separated by 10% SDS-PAGE and transferred to nitrocellulose membranes. Then Total-GSK-3β (T-GSK-3β) (Proteintech), phosphorylated GSK-3β (P-GSK-3β) (Proteintech) and Total-AKT1 (T-AKT1) proteins were determined by Western blotting.

### Statistical Analysis

The results were analyzed by the statistical software GraphPad Prism (version 8.0.1, GraphPad Software Inc., San Diego, CA, United States), expressed as mean ± standard deviation. The inter-group comparison was performed by one-way ANOVA with Tukey’s post-hoc test or Kruskal–Wallis ANOVA with post-hoc Dunn’s multiple comparison tests. *p* < 0.05 was considered statistically significant.

## Results

### Common Targets of Resveratrol, Sympathetic Stress, and Myocardial Inflammation Screened With Network Pharmacology

**FIGURE 1:**
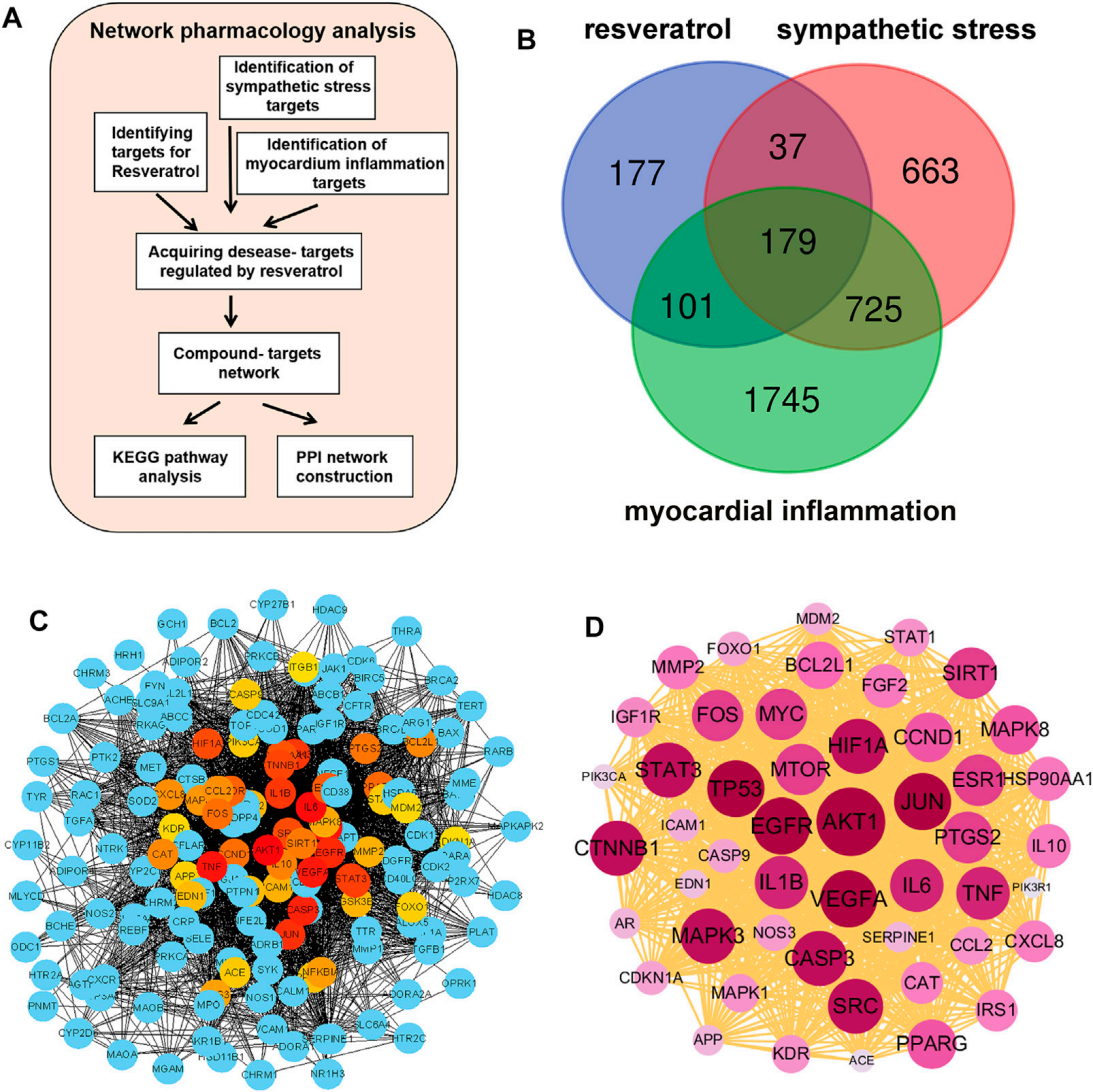
Screening of common targets based on network pharmacology. **(A)** Flow chart of network pharmacology prediction. **(B)** Sympathetic stress targets and myocarditis targets were collected from the genecards database, and resveratrol targets were collected from the TCMSP database, SWISS target prediction database, SEA database, and QSAR database. After the intersection of the Wayne diagram, a total of 179 targets were obtained. **(C)** Protein-protein interaction analysis (PPI) was performed for the 179 common targets. **(D)** According to the degree value, the PPI was constructed using the top 50 targets.

The flow chart briefly describes the process of network pharmacology screening targets and analysis (Figure 1A). Based on the drug structure similarity algorithm and data mining, we screened out 494 potential candidate targets of resveratrol with different databases. Using the Genecards database, 1,604 sympathetic stress targets and 2,750 myocardial inflammation targets were obtained respectively. A total of 179 common targets were obtained as shown in the Venn diagram (Figure 1B). The common targets were put into the string database for analysis and obtain the PPI network diagram (Figure 1C). The classical inflammatory molecules IL-1β and IL-6 were among the core targets, as well as SIRT1, the well-known target of resveratrol (Figure 1C; Supplementary Figure S1). Then, according to the degree value, we further analyzed the top 50 targets by the PPI network, in which AKT1 ranks the core targets (Figure 1D).

### AKT1 Significantly Correlated Target of Resveratrol in Improving Acute Sympathetic Stress-Induced Heart Injury

**FIGURE 2:**
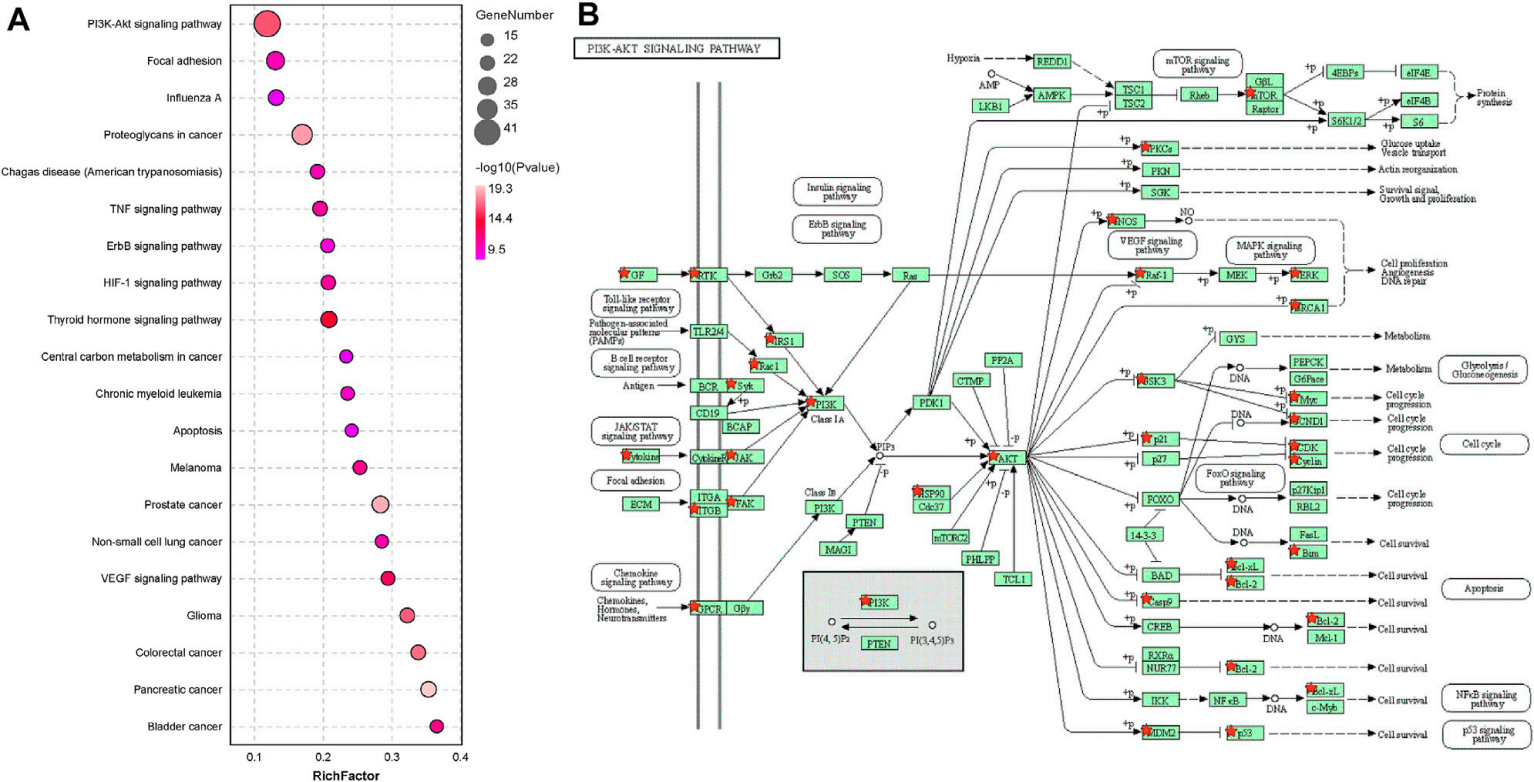
AKT1 is a significantly correlated target. **(A)** KEGG pathway analysis of 179 targets (Top 20 targets of KEGG enrichment). **(B)** KEGG pathway analysis of the PI3K/AKT pathway.

In the KEGG pathway analysis of the common genes, the “PI3K/AKT pathway” was among the top enriched pathways (Figure 2A). The targets are indicated on the map of the “PI3K/AKT pathway”, which shows that AKT may be a critical link in the pathway and AKT1 has the closest relationship with other targets. (Figure 2B).

### Resveratrol Inhibited Isoproterenol-Induced Phosphorylation of AKT1 in Cardiomyocytes

**FIGURE 3:**
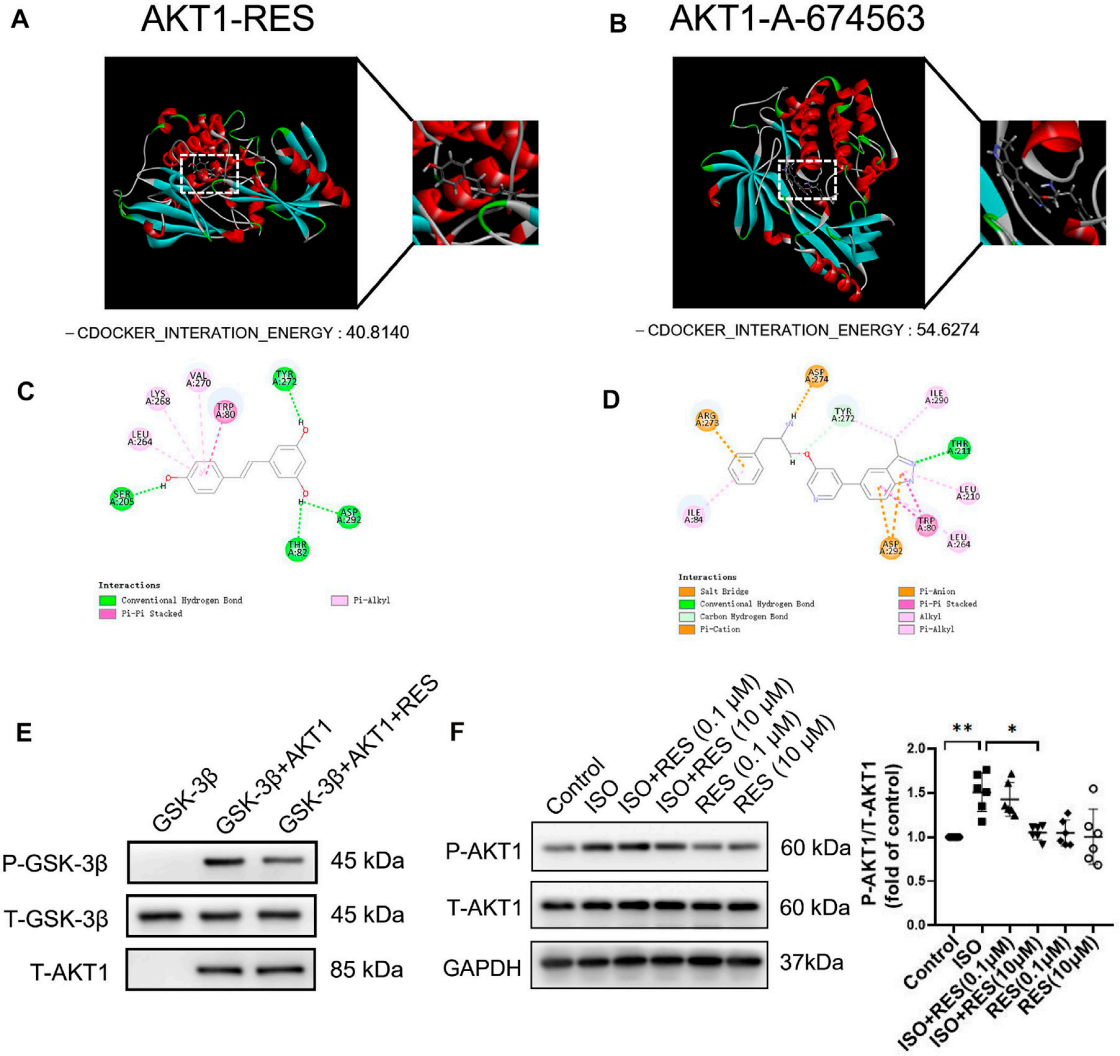
Resveratrol improves the molecular mechanism of acute sympathetic stress-induced heart injury through AKT1. **(A)** Three-dimensional docking of resveratrol and AKT1. **(B)** Three-dimensional docking of A-674563 and AKT1. **(C)** Two-dimensional docking of resveratrol and AKT1. **(D)** Two-dimensional coupling of A-674563 and AKT1. **(E)** AKT1 activity in the *in vitro* kinase assay. Recombinant active AKT1, purified GSK-3β and RES were added into the kinase buffer as indicated. The AKT1 kinase activity was indicated by the phosphorylation of GSK-3β. **(F)** NMCMs were exposed to isoproterenol (10 μmol/L) for 1 h with or without resveratrol (100 nmol/L) or (10 μmol/L) pretreatment for 30 min. Total-AKT1 (T-AKT1) and phosphorylated AKT1 (P-AKT1) protein levels were detected by Western blot. *n* = 6; ^*^ *p* < 0.05; ^**^ *p* < 0.01; ^***^ *p* < 0.001; RES, Resveratrol; ISO, isoproterenol. Data are mean ± SD (Kruskal–Wallis ANOVA with *post-hoc* Dunn’s multiple comparison tests).

We evaluated the interaction between resveratrol and AKT1 (PDB ID: 4EJN) by molecular docking (Figure 3A). The docking results show that The -CDOCKER_INTERATION_ENERGY of resveratrol and AKT1 was 40.8140 (Figure 3A), The -CDOCKER_INTERATION_ENERGY of resveratrol and A-674563(AKT1 inhibitor) was 54.6274 (Figure 3B). The results suggested that resveratrol can bind to AKT1 with similar energy as the AKT1 inhibitor, A-674563. 2D molecular docking results show that resveratrol produces conventional hydrogen bonds with SER205, THR82, ASP292 and TYR272 of AKT1, a Pi-alkyl interaction with LEU264, LYS268, and VAL270, and a Pi-Pi stacked interaction with TRP80 (Figure 3C). To confirm the direct effect of resveratrol on AKT1 kinase activity, we perform the *in vitro* kinase assay. Resveratrol significantly decreased the phosphorylation of GSK-3β by recombinant AKT1 kinase (Figure 3E). The inhibitory effect of RES on AKT1 was further determined in cardiomyocytes. We pretreated NMCMs with resveratrol for 30 min followed by isoproterenol treatment (10 μmol/l) for 1 h. Western blotting revealed that the protein expression of phosphorylated AKT1 increased significantly after isoproterenol treatment, while resveratrol significantly inhibited AKT1 phosphorylation (Figure 3F), and the protein expression of total AKT1 remained essentially unchanged (Supplementary Figure S2). We further pretreated NMCMs with an antagonist of β-adrenoceptor, propranolol (100 nmol/l) or an inhibitor of protein kinase A, PKI (10 μmol/l) for 1 h before isoproterenol treatment. Western blotting revealed that both propranolol and PKI significantly decreased the phosphorylation of AKT1 induced by isoproterenol (Supplementary Figure S3). Thus, resveratrol can inhibit acute sympathetic stress-induced phosphorylation of AKT1 in cardiomyocytes.

### AKT1-Mediated Activation of the NLR Family, Pyrin Domain Containing 3 Inflammasome in NMCMs After Acute Sympathetic Stress

**FIGURE 4:**
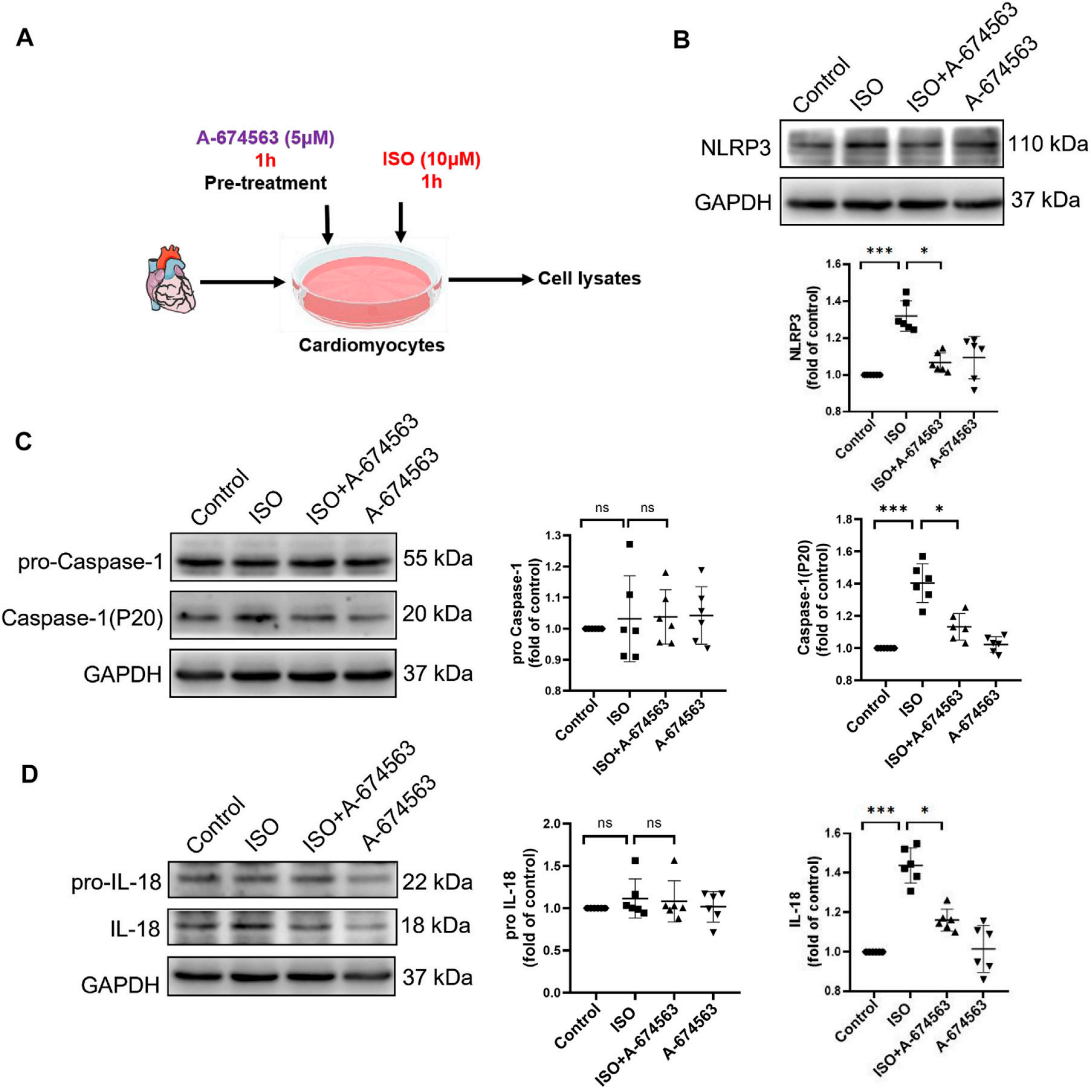
A-674563 inhibits isoproterenol-induced activation of the NLRP3 inflammasome in NMCM. NMCMs were exposed to isoproterenol (10 μmol/L) for 1 h with or without A-674563 (5 μmol/L) pretreatment for 1 h. **(A)** Mode in NMCMs. **(B–D)** The protein levels of NLRP3, pro-caspase-1, caspase-1(P20), pro-IL-18, and cleaved IL-18 were detected by western blotting. *n* = 6; ^*^ *p* < 0.05; ^**^ *p* < 0.01; ^***^ *p* < 0.001; RES, Resveratrol; ISO, isoproterenol. Data are mean ± SD (Kruskal-Wallis ANOVA with *post-hoc* Dunn’s multiple comparison tests).

To determine the role of AKT1 in isoproterenol-induced NLRP3 inflammasome activation, we pretreated NMCMs with AKT1 inhibitor A-674563 (5 μmol/l) for 1 h before isoproterenol treatment (Figure 4A). Western blotting revealed that protein levels of NLRP3 (Figure 4B), P20 (Figure 4C), and cleaved IL-18 (Figure 4D) were increased with isoproterenol treatment, but could be suppressed with pretreatment with A-674563. Thus, the phosphorylation of AKT1 is the upstream molecule regulating NLRP3 inflammasome activation in cardiomyocytes under sympathetic stress.

### Resveratrol Alleviates Isoproterenol-Induced Inflammasome Activation in Cardiomyocytes

**FIGURE 5:**
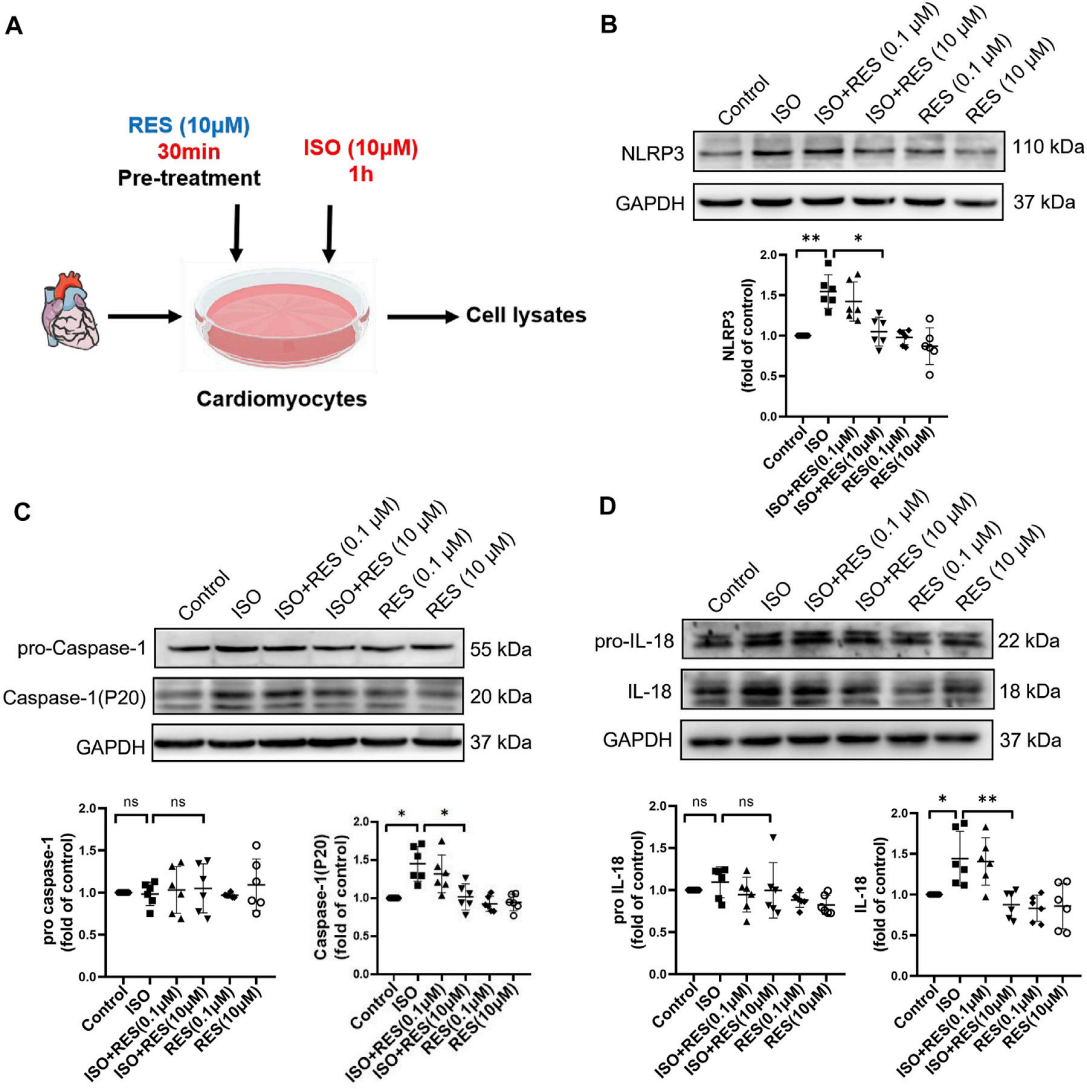
Resveratrol inhibits isoproterenol-induced NLRP3 inflammasome activation in NMCM. NMCMs were exposed to isoproterenol (10 μmol L^−1^) for 1 h with or without resveratrol (100 nmol/l) or (10 μmol/l) pretreatment for 30 min. **(A)** Mode in NMCMs. **(B–D)** The protein levels of NLRP3, pro-caspase-1, caspase-1(P20), pro-IL-18, and cleaved IL-18 were detected by western blotting. *n* = 6; ^*^ *p* < 0.05; ^**^ *p* < 0.01; ^***^ *p* < 0.001; RES, Resveratrol; ISO, isoproterenol. Data are mean ± SD (Kruskal-Wallis ANOVA with post-hoc Dunn’s multiple comparison tests).

As shown in the mode diagram in Figure 5A, NMCMs were exposed to isoproterenol for 1 h with or without resveratrol pretreatment for 30 min. The protein expression of NLRP3, P20, and cleaved-IL-18 on NMCMs increased significantly after 1 h of isoproterenol treatment. Pretreatment with resveratrol significantly inhibited the increase in NLRP3, P20, and cleaved-IL-18 induced by isoproterenol (Figures 5B–D). These results suggested that resveratrol reduces isoproterenol-induced inflammasome activation in cardiomyocytes.

### Resveratrol Pretreatment Alleviates Isoproterenol-Induced Cardiac Inflammation

**FIGURE 6:**
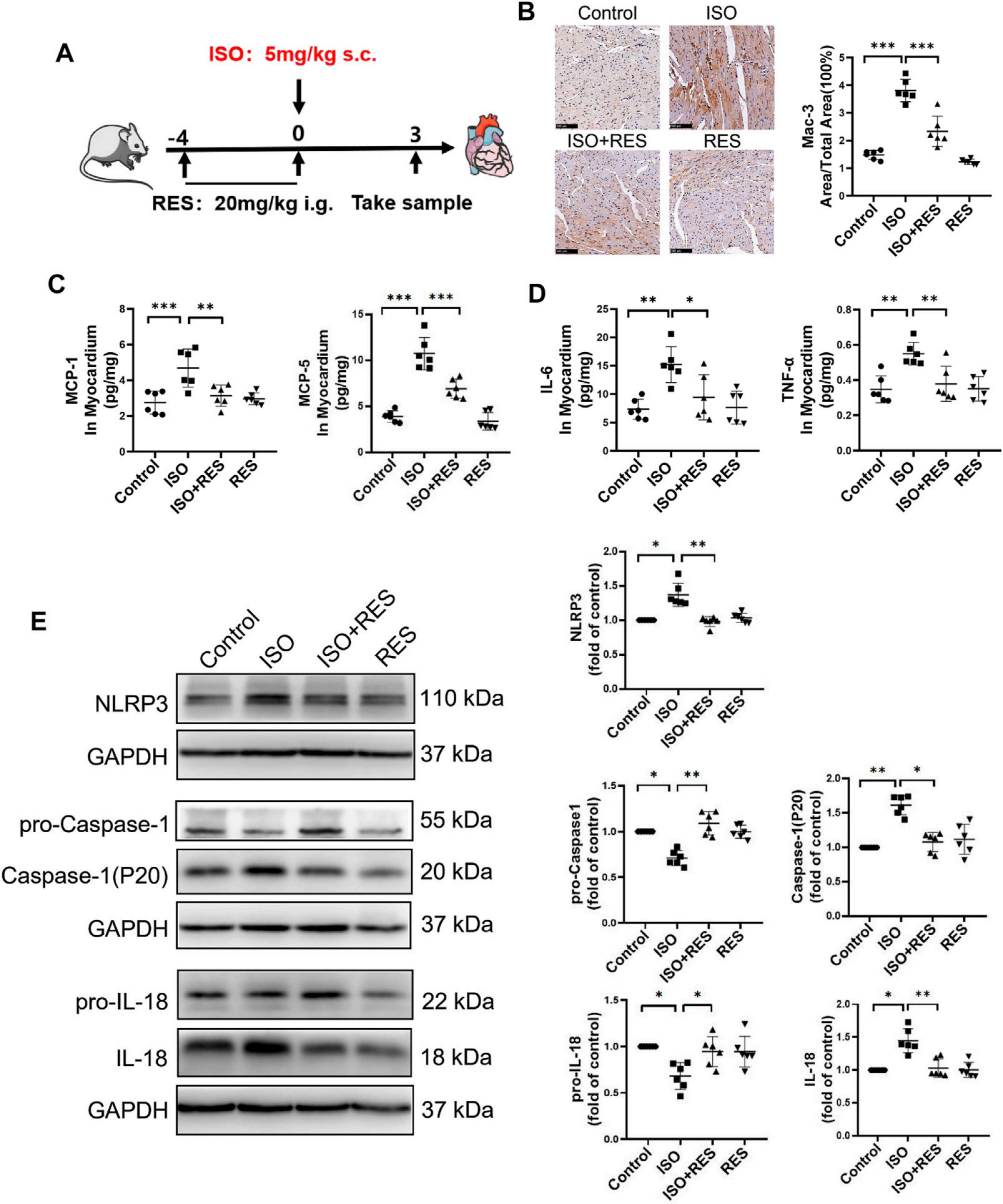
Resveratrol alleviated isoproterenol-induced cardiac inflammation and activation of the NLRP3 inflammasome in mice. Ten-week-old male C57BL/6J mice were administered resveratrol (20 mg/(kg⋅d) body weight, intragastrically) for 3 days prior to isoproterenol (5 mg/kg body weight, subcutaneously) treatment. **(A)** Mode in mice. **(B)** Representative images and quantification of immunostaining for Mac-3 (a macrophage marker) in the heart on the third day after isoproterenol treatment (*n* = 6; scale bars: 100 μm). **(C–D)** Concentrations of indicative chemokines (MCP-1, MCP-5) and pro-inflammatory cytokines (IL-6 and TNF-α) on the 3rd day after ISO treatment as measured by ELISA (*n* = 6). **(E)** Protein levels of NLRP3, pro-caspase-1, caspase-1(P20), pro-IL-18 and cleaved IL-18 were detected by Western blotting. ^*^ *p* < 0.05; ^**^ *p* < 0.01; ^***^ *p* < 0.001; RES, Resveratrol; ISO, isoproterenol. Data are mean ± SD (one-way ANOVA with Tukey’s *post-hoc* test or Kruskal–Wallis ANOVA with *post-hoc* Dunn’s multiple comparison tests).

As shown in Figure 6A, male, 10 week-old C57BL/6J mice were pretreated by intragastric administration of resveratrol (20 mg/(kg•d)) for four consecutive days, and then isoproterenol (5 mg/kg) was injected subcutaneously for a single time. The isoproterenol-induced cardiac inflammasome activation can be inhibited by resveratrol pretreatment. MAC-3 was further stained to evaluate macrophage infiltration in heart tissue. The results showed that the positive area of MAC-3 in the mouse heart was increased significantly on the third day after isoproterenol injection. Instead, pretreatment with resveratrol significantly inhibited isoproterenol-induced cardiac macrophage infiltration (Figure 6B). Consistently, ELISA analysis revealed that the expression of macrophage chemokines MCP-1 and MCP-5 (Figure 6C), as well as proinflammatory cytokines IL-6 and TNF-α, were upregulated in the isoproterenol-treated group, which were inhibited by resveratrol pretreatment (Figure 6D). Moreover, the isoproterenol-induced NLRP3 inflammasome activation was suppressed by resveratrol (Figure 6E). Thus, resveratrol inhibited isoproterenol-induced cardiac inflammation.

## Discussion

**FIGURE 7:**
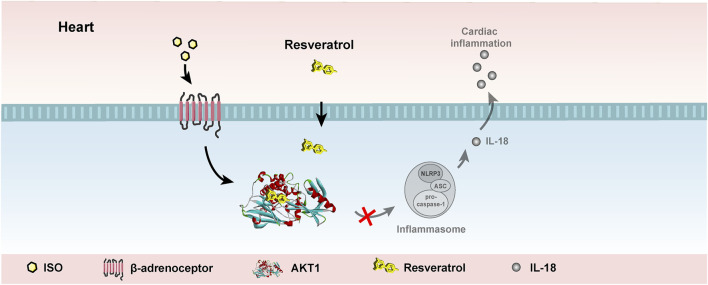
Working model. Resveratrol suppresses acute sympathetic stress-induced inflammasome activation in cardiomyocytes *via* inhibiting AKT1.

The present study found that AKT1 phosphorylation is the upstream mechanism that promotes activation of the NLRP3 inflammasome in cardiomyocytes after isoproterenol treatment. Resveratrol can target AKT1 to inhibit its phosphorylation by isoproterenol treatment, resulting in suppression of inflammasome activation in cardiomyocytes and cardiac inflammation (Figure 7). AKT1 is an important target for the anti-inflammatory role of resveratrol in the heart.

AKT1 is an intracellular kinase, it plays a central role in the regulation of metabolism, homeostasis, survival, and proliferation (O'Neill and Abel, 2005). Whereas, there is no clear information on the role of AKT1 in isoproterenol-induced cardiac inflammasome activation. In the present study, we found that isoproterenol induced AKT1 phosphorylation. Treatment with a selective inhibitor of AKT1 inhibited the activation of NLRP3 inflammasome in cardiomyocytes following isoproterenol treatment. Thus, AKT1 phosphorylation is involved in the isoproterenol-induced cardiac inflammasome activation. AKT isotypes are shown to play a different role in inflammation in different tissues and cells. AKT1 in the vasculature promotes acute inflammatory responses by increasing microvascular leakage (Di Lorenzo et al., 2009); while AKT2 activity in neutrophils is important for neutrophil adhesion during vascular inflammation (Li et al., 2014). The downregulation of the AKT1-MTOR-RPS6KB1 pathway was also shown to trigger mitophagy in macrophages, which reversed mitochondrial membrane potential collapse and inhibit NLRP3 inflammasome activation (Guo W et al., 2014). The present study shows that in the heart under sympathetic stress, the isotype of AKT1 may be an important target for the intervention of inflammation activation and initiation of inflammation.

Sympathetic stress is an important factor in the promotion of the progression of cardiovascular disease (Burnstock, 2017). Excessive activation of the sympathetic nervous system causes cardiac inflammation leading to myocardial injury. During the process, NLRP3 inflammasome activation plays a critical role in cardiac inflammation by promoting IL-18 activation (Xiao et al., 2018). Blocking IL-18 with neutralizing antibodies at 1 h, but not later, alleviated isoproterenol-induced cardiac inflammation, suggesting that early activation of the NLRP3 inflammasome is critical for the initiation of cardiac inflammation under sympathetic stress (Xiao et al., 2018). However, interventions targeting early inflammasome activation are limited. The present study found that resveratrol can target AKT1 to inhibit early activation of the inflammasome. Thus resveratrol can be a potential strategy to suppress cardiac inflammation at the initiation phase under sympathetic stress.

The clinical trials on resveratrol often show conflicting results, but a higher dose of resveratrol (>500 mg/d) has shown promising anti-inflammatory activity (Gorabi et al., 2021). It may be partly because resveratrol can act on various targets. Regarding the anti-inflammatory effects of resveratrol (Baur and Sindair, 2006), the PI3K pathway (Li et al., 2021), and the SIRT1/NRF2 signaling pathway (Nie et al., 2020) are also involved. It was previously reported that SIRT1 activation inhibited ROS-associated NLRP3 inflammasome activation and ameliorates cardiac pyroptosis during myocardial I/R injury through Akt-PDH signaling pathway (Han Y et al., 2020). Therefore, clarifying critical targets under specific pathologic conditions can be helpful in determining the underlying mechanism of contrasting outcomes. In recent years, with the emergence of new disciplines such as network pharmacology and multidirectional pharmacology, scientists have recognized and explored new active mechanisms of existing drugs (Guney et al., 2016). In the present study, AKT1 ranks first among the predicted targets of network pharmacology, suggesting its critical role in the anti-inflammatory activity on the heart, especially under sympathetic stress. However, this interpretation is based on cellular and animal experiments; the exact relationships between resveratrol, cardiac inflammation, and sympathetic stress need to be evaluated in future clinical trials.

In conclusion, resveratrol can inhibit AKT1 phosphorylation in cardiomyocytes following isoproterenol treatment, thus resulting in the suppression of the activation of the NLRP3 inflammasome and cardiac inflammation. AKT1 is a promising potential target for the treatment of cardiac inflammatory injury under sympathetic stress.

## Data Availability

The original contributions presented in the study are included in the article/Supplementary Materials, further inquiries can be directed to the corresponding authors.
