# Supplementary material for: Resveratrol Targets AKT1 to Inhibit Inflammasome Activation in Cardiomyocytes Under Acute Sympathetic Stress
Source: Front Pharmacol. 2022 Feb 17;13:818127. doi: 10.3389/fphar.2022.818127 (PMC8891986; doi:10.3389/fphar.2022.818127)
Supplement: Supplementary file 1 [file DataSheet1.docx]

**Supplementary information**


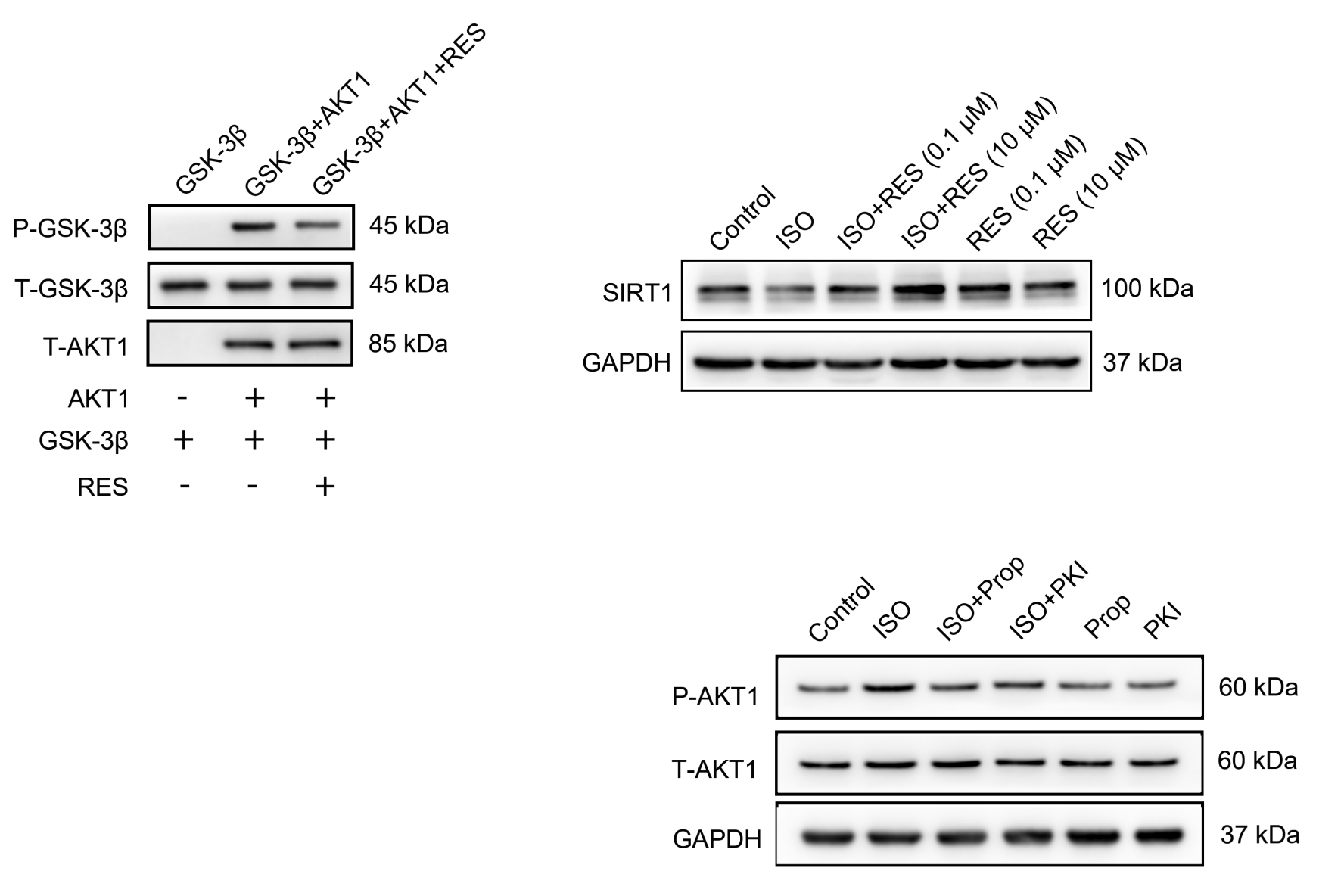

**Figure S1. RES reversed ISO-induced SIRT1 depression in cardiomyocytes.** NMCMs were exposed to isoproterenol (10 μmol/L) for 1 hour with or without resveratrol (100 nmol/L) or (10 μmol/L) pretreatment for 30 minutes. SIRT1 protein levels was detected by Western blot. n=6; ^*^*P*<0.05; ^**^*P*<0.01; ^***^*P*<0.001; RES, Resveratrol; ISO, isoproterenol. Data are mean ± SD (Kruskal-Wallis ANOVA with post-hoc Dunn’s multiple comparison tests).

**Figure S2. the AKT1 expression levels in cardiomyocytes following ISO and RES treatments.** NMCMs were exposed to isoproterenol (10 μmol/L) for 1 hour with or without resveratrol (100 nmol/L) or (10 μmol/L) pretreatment for 30 minutes. Total-AKT1 (T-AKT1) protein levels were detected by Western blot. n=6; ^*^*P*<0.05; ^**^*P*<0.01; ^***^*P*<0.001; RES, Resveratrol; ISO, isoproterenol. Data are mean ± SD (Kruskal-Wallis ANOVA with post-hoc Dunn’s multiple comparison tests).


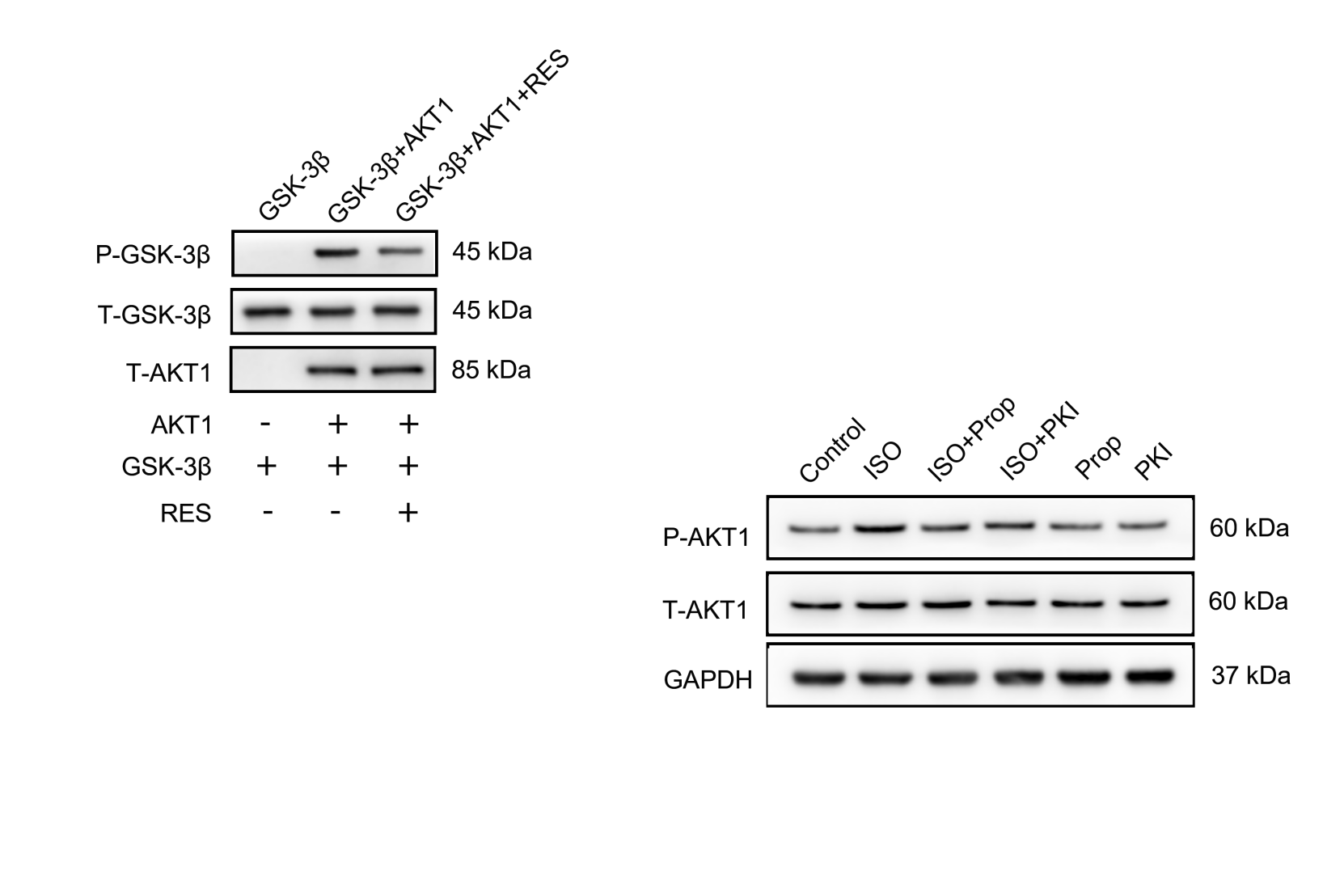


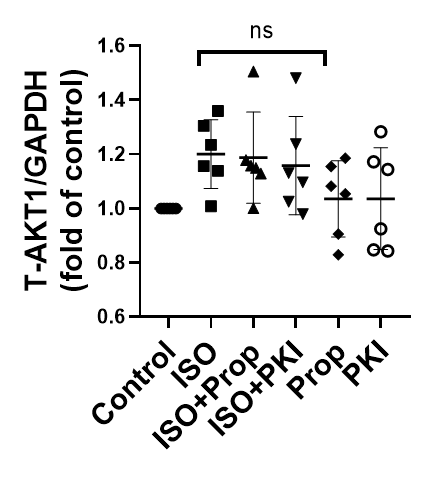


**Figure S3. Propranolol and PKI inhibit ISO-induced AKT1 phosphorylation in cardiomyocytes.** NMCMs were exposed to isoproterenol (10 μmol/L) for 1 hour with or without Propranolol (100 nmol/L) and PKI (10 μmol/L) pretreatment for 1 hour. Total-AKT1 (T-AKT1) and phosphorylated AKT1 (P-AKT1) protein levels were detected by Western blot. n=6; ^*^*P*<0.05; ^**^*P*<0.01; ^***^*P*<0.001; Prop, Propranolol; PKI, PKA inhibitor. Data are mean ± SD (Kruskal-Wallis ANOVA with post-hoc Dunn’s multiple comparison tests).
